# Probing the molecular design of hyper-branched aryl polyesters towards lubricant applications

**DOI:** 10.1038/srep18624

**Published:** 2016-01-05

**Authors:** Joshua W. Robinson, Yan Zhou, Priyanka Bhattacharya, Robert Erck, Jun Qu, J. Timothy Bays, Lelia Cosimbescu

**Affiliations:** 1 Pacific Northwest National Laboratory, Richland, Washington 99352; 2 Oak Ridge National Laboratory, Oak Ridge, Tennessee 37831; 3 Argonne National Laboratory, Lemont, Illinois 60439.

## Abstract

We report novel polymeric materials that may be used as viscosity index improvers (VII) for lubricant applications. Our efforts included probing the comb-burst hyper-branched aryl polyester architecture for beneficial viscosity and friction behavior when utilized as an additive in a group I oil. The monomer was designed as to undergo polymerization via polycondensation within the architectural construct (AB_2_), typical of hyperbranched polymers. The monomer design was comprised of aliphatic arms (12 or 16 methylenes) to provide the necessary lipophilicity to achieve solubility in a non-polar medium. Once polymerized, via catalyst and heat, the surface alcohols were functionalized with fatty acids (lauric and palmitic). Controlling the aliphatic nature of the internal arms and peripheral end-groups provided four unique flexible polymer designs. Changing the reaction time and concentration provided opportunities to investigate the influence of molecular weight and branching density on oil-solubility, viscosity, and friction. Oil-solubility was found to decrease with fewer internal carbons, but the number of internal carbons appears to have little influence on the bulk solution viscosity. At concentrations of 2 wt % in a group I base oil, these polymer additives demonstrated an improved viscosity index and reduced friction coefficient, validating the basic approach.

The main driving force behind the development of new lubricant additives is to meet ever increasing challenges towards fuel economy and environmental stewardship. Lubricant formulations include many additives to improve efficiency (i.e. reduce friction) and prolong the life of mechanical components (i.e. reduce wear)^1^. Polymers constitute a significant portion of the additives blended into lubricants. Their major role as a viscosity index improver (VII) in this concerted ballet of additives is to mitigate viscosity changes that the lubricant experiences over operating temperatures (e.g. −20 to 150 °C). Much emphasis has been placed on reducing the natural thinning effect that occurs at higher temperatures (40 to 150 °C). The ideal polymeric additive would have minimal influence on the lubricant’s viscosity at low temperatures (−20 to 40 °C) and significantly reduce the natural thinning effect of the lubricant at elevated temperatures (40 to 150 °C). High performing polymeric additives become even more imperative as original equipment manufacturers (OEMs) design engines that show significant fuel economy gains when utilizing low viscosity oils (e.g. SAE 0 W-20)^2^,^3^. Without polymeric and anti-friction additives, the neat oil’s lubricity plummets at high temperatures causing greater boundary friction and wear leading to fuel efficiency losses and possible mechanical failure. Therefore, engine durability requires lubricant additives that help the oil to retain oil viscosity at elevated temperatures.

Current viscosity modifiers include polymers that have high molecular weights (>100 kDa) such as olefin copolymers (OCP; e.g. poly(ethylene)(propylene), lipophilic poly(styrene)(diene), etc.) and polyesters with fatty side chains (e.g. poly(alkyl methacrylate) (PAMA), where the alkyl side-chain contains 8–30 saturated carbons)^4^. Historically, a linear architecture with elastomeric features was exploited towards the viscosity modification of group I to group III oils. These earlier designs are typically prone to polymeric degradation caused by high shear forces within an internal combustion engine decreasing their effectiveness as viscosity modifiers^5^. Modern designs include comb^6^, star^7^,^8^, and branched^9^ architectures which are more robust against shear degradation. Various polymeric architectures are depicted in Fig. 1. However, dendritic-like polymers (i.e. hyper-branched, dendrimers, and linear polymers with dendritic side chains) as lubricant viscosity modifiers have not been described in the literature, although they are believed to be more resilient, versus linear architectures, against shear degradation^9^. Presumably, the lack of publications in this area may be due to cost, limitations in the synthesis, and general realization that rigid and highly branched structures modify the viscosity of solutions to a lesser extent than do their linear counterparts with similar molecular weights. In fact, new mathematical theories for intrinsic viscosity had to be developed for non-linear architectures to provide accurate molecular weight calculations which in turn provide additional insight into structural features^10^. In general, the average hydrodynamic volume (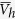) of a non-linear polymer is smaller than its linear counterpart (i.e. random coil) with a similar molecular weight in an ideal solvent. In addition, rigid and highly branched polymeric structures (i.e. dendrimers) undergo a minimal interaction, caused by chain entanglement, with one another in solution^11^,^12^. The lack of intermolecular entanglement has afforded such dendritic polymers to be utilized as rheology modifiers in polymeric melt-blends and receive colorful descriptions such as “Molecular Ball-Bearings”^13^. Overall, molecular weight and architecture influence the levels of chain entanglement and 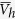 at given concentrations, which contribute to the modification of lubricant viscosities when polymeric additives are employed.

Flexible hyper-branched polymers in base oils may provide alternative benefits yet achieved towards improving the performance of lubricant formulations. Presumably, in addition to improving the viscosity index of the oil, they should have longer life due to a structure that is more resistant to shear degradation, provide dynamic surface interaction via tailoring of peripherals and architecture, undergo a more significant change in 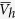 as related to changes in solubility governed by temperature, and limit increases to viscosity of the base oil at relatively low temperatures. We envision a non-linear polymeric additive increasing lubricity via two pathways: 1) mitigation of viscosity changes of base oils over a range of temperatures and 2) reduce friction in the boundary and mixed lubrication regimes at the contacting surface asperities.

The viscosity index (VI) number of an oil provides insight into the thinning effect (decrease in viscosity) of a lubricant formulation at temperatures of 40 and 100 °C (ASTM D2270). Ideally, the decrease in viscosity would be minimal between these temperatures, affording consistent lubricity. However, a natural thinning effect is observed and polymeric additives provide an opportunity to minimize the loss in viscosity at elevated temperatures. This is achieved either by increasing the shear thickness (intermolecular interactions) of the polymer/oil blend and/or by a globular-to-coil transition (expansion) of the polymer in the oil between low to high temperatures (−20 to 150 °C). Viscosity modifiers (VM) generally have chemical compositions that are similar to the oil (saturated carbons) and therefore are highly soluble over a wide range of temperatures. Viscosity index improvers (VII) generally include less soluble components in which overall solubility increases with temperature providing a more significant change in hydrodynamic volume (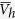) from 40 to 100 °C^14^. VIIs are theorized to undergo the globular-to-coil transition due to their relatively lower shear thickening effect at lower temperatures, as compared to VMs, while reducing shear thinning at higher temperatures thereby providing competitive to superior viscosity index (VI) numbers. Depending on degree of branching, chemical composition, and polymer-solvent interaction, hyper-branched polymers to dendrimers have been described to exist in a globular state which may undergo a significant conformational change dependent on temperature and solubility influences providing an opportunity to exploit them as VIIs^15^.

Hyper-branched polymers may be prepared via an AB_x_ or AB_x_ + B_y_ approach (x ≥ 2, y ≥ 3). In general, the monomer is composed of two types of functional groups (A and B) that will exclusively react with one another. These are one-pot polymerizations which encompass step and chain growth synthetic approaches such as polycondensation, self-condensing vinyl polymerization (SCVP), and ring-opening multi-branching polymerization (ROM-BP). The literature describes a myriad of flexible to rigid structures that cover a landscape of polar functionalities (e.g. amines, amides, ethers, esters, aromatics, etc.)^16^. A few examples of non-polar or even lipophilic hyper-branched polymers have been published^17^. Additional structural variations may be incorporated by varying the core density, or average degree of branching (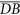), as well as tailoring the peripheral functional groups. Overall, the plethora of synthesized hyper-branched polymers is matched only by the diversity in application-driven investigations.

Application-motivated investigations of hyper-branched polymers have included biomedical devices (e.g. sensors, drug delivery, etc.), rheology modification (i.e. in the melt, solution properties), emulsifiers, as stress modulus additives (coating additives), and energy materials (e.g. solar panels, energy storage electrolyte, etc.)^16^,^18^. In particular, Hawker and co-workers prepared hyper-branched poly(ethylene glycol)s towards their investigations of ion-conducting materials^19^. They varied the length of the ethylene glycol oligomer (n = 1, 2, 5) on a 3,5-dioxybenzoate branching unit which they polymerized via a polycondensation protocol affording an ion conducting comb-burst hyper-branched aryl polyester. For our initial study utilizing hyper-branched polymers as lubricant additives, the flexible oligomeric units were attractive for solubility and conformational changes as well as the aromatic branching was attractive for thermo-stability reasons. However, for our purposes we modified their approach to include lipophilic oligomers within the core structure and post-functionalized the peripheral alcohols with fatty acids to increase conformational changes and chain entanglement opportunities in the corona, as well as modify the lipophilicity and therefore the solubility of the resulting compounds in base oils. In the following sections we disclose the preparation of a comb-burst hyper-branched aryl polyester (HAPe) with various internal and external lipophilic groups, molecular weights, and architectural features (i.e. 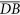). This afforded the opportunity to probe the structural features of HAPe in relationship to viscosity and friction modification at 2 wt % in group I oils.

## Results

### Preparation and characterization of polymers

**Figure 2:**
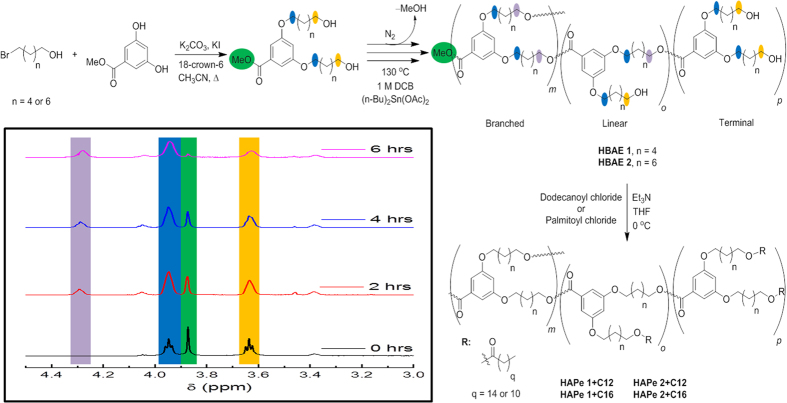
Synthetic route towards novel comb-burst hyper-branched aryl polyesters (HAPe), subsequent post-modification, and stacked ^1^H-NMR spectrum illustrating progression of polymerization.

We targeted four derivatives of hyper-branched aryl polyesters (HAPe). This included variation of the lipophilic linkage of the monomer by six or eight carbons and the modification of the subsequent polymer with long chain acyl chlorides (dodecanoyl chloride, C12, or palmitoyl chloride, C16), to afford saturated fatty esters in the corona (Fig. 2). The three-step synthesis included a quantitative preparation of the monomer^20^, a gravimetric yield greater than 50% of the alcohol-functionalized polymerization product, followed by a variable yield (49% to quantitative) post-functionalization of the peripheral alcohols into fatty esters^21^. Over-polymerization (leading to an insoluble gel) occurred quite readily with our monomer while utilizing the procedure described by Hawker and co-workers towards their polyethylene glycol (PEG) analog^19^. To mitigate the over-polymerization of our analog, as well as reduce the average degree of branching, the monomer was diluted in dichlorbenzene (DCB). Thus a 1.0 M solution of the monomer was heated near reflux and treated with a tin catalyst (*n*-Bu_2_SnOAc_2_) to initiate polymerization. Although more controllable than the neat polymerization, the solution polymerization was not free of challenges, as to prevent over-polymerization. The polymerization had to be monitored closely by ^1^H NMR to prevent the formation of an insoluble resin which appeared to occur with relatively high average degree of polymerizations (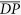). The stacked ^1^H NMR spectra inset in Fig. 2 illustrates the typical progression of these step-growth polymerizations over 6 hours, which was generally the time required to achieve a soluble polymer. The broadening and slow disappearance of the methyl ester peak at 3.88 ppm (green) was closely monitored and utilized in conjunction with the ether methylene peak at 3.95 ppm (blue) to target 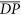
s between 10 and 30. As is the case with step-growth polymerizations, the relationship of the conversion of the monomer into polymer with molecular weight is non-linear. Therefore, the determination of 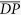 via an aliquot removed from the reaction mixture and measured by ^1^H NMR did not provide accurate insight into the progression of the polymerization in regards to molecular weight. To determine number-average molecular weight (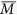
_*n*_) via ^1^H NMR spectroscopy, analogs 1, 2, 3, and 4 were first purified by precipitation. Polymerizations 5, 6, and 7 were terminated by simply adding the next step reagents, i.e. palmitoyl chloride (C16) and triethyl amine (TEA). Analogs 1, 2, 3, and 4 were subsequently treated with either dodecanoyl chloride (C12) or C16 and TEA in tetrahydrofuran (THF). All analogs were then purified via precipitation techniques prior to further characterization.

The polymers were analyzed by nuclear magnetic resonance (NMR) spectroscopy to determine 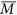
_*n*_ and chemical composition (i.e. percent of post-functionalization, P_f_%). The 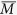
_*n*_s were found to range from 3.05 to 34.5 kg/mol (Table 1). The P_f_% was determined to be between 49 to 99%. The apparent number-average molecular weights (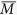
_*n*_^app^) and molar dispersity (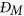) were determined to be between 4.75−18.2 kg/mol and 1.55−3.27, respectively, by size exclusion chromatography (SEC). There are discrepancies between ^1^H NMR and SEC 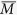
_*n*_s suggesting an error in calculating 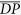 via end group analysis or the hydrodynamic volume of polymers with comparable molar masses have significantly different architectures (linear vs. dendritic) thereby influencing the elution volume. For example, analogs 1 and 2 were determined by end group analysis to have 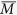
_*n*_s approximately half of analogs 3 and 4. However, SEC suggests the reverse understanding with analogs 1 and 2 having 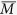
_*n*_^app^s greater than analogs 3 and 4. Complications in obtaining accurate molar masses from ^1^H NMR are due to increasing peak overlap between the broadening methylene ether (δ = 3.95) and gradual disappearance of the methyl ester (δ = 3.88) protons. However, the broad (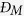 < 3.3) and multi-modal spectra obtained from SEC also limits accurate interpretation of how the hydrodynamic volume (*V*_*h*_) may be related to architectural or molecular weight differences. The elucidation of the architectural differences of these polymeric samples, by determining the average degree of branching (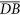) via NMR spectroscopy, was not possible. Similar systems consist of aromatic protons which are close to the alcohol/ester functionality in the polymer^22^,^23^ and therefore respond to those respective environmental changes in the molecule. The 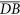 can rather easily be determined from integration of the phenyl protons. In our case however, all functional groups that undergo a change are six or eight carbons away from the phenyl ring, and thus the aromatic protons do not “see” a responsiveness to these changes. Although we are unable to confirm the branched structure by NMR analysis, the AB_2_ polycondensation reaction is well-known to produce hyper-branched architectures.

### Lubricant Performance

Our primary goal was to test the performance of these polymers/oligomers as viscosity index improvers. In addition, the relatively high density of heteroatoms was expected to increase the interaction with the metal surfaces, thereby reducing friction near the boundary regime. The molecular feature that is responsible for friction reduction results in a reduced lipophilicity and therefore solubility in base oils. These blends, after long periods of agitation, formed turbid mixtures at room temperature. Additional heating (≥50 °C) was required to prepare homogeneous blends which underwent a reversible phase separation at temperatures <50 °C. Although phase separation of HAPes from neat base oils <50 °C is not ideal, it is expected that the miscibility would increase within a formulated package which includes various other additives that may promote solubility. The polymers were mixed in group I oil (ExxonMobil) at concentrations of 1−3 wt % via agitation and heating. Blends of benchmarks (Bench.) 1 and 2 were prepared at the appropriate concentrations and utilized as comparative model systems due to their prevalence as viscosity modifiers within the lubricant field. In order to calculate the viscosity index of the additives, the dynamic viscosity (centipoise; cP) of the 2 wt % blends was measured at 40 and 100 °C utilizing a rotary spindle viscometer. Kinematic viscosity values, in centistokes (cSt = cP/ρ; where ρ = g/mL), were calculated from these numbers and are reported in Table 2 at a 2 wt % concentration. Viscosity indices (VI) were generated by an online calculator widely accepted by experts in the field^24^. Another significant value for lubricant evaluation and the subsequent determination of the oil grade it falls in^25^, is a high temperature high shear test (HTHS), which imposes a low viscosity limit on the final oil^3^. This test is performed at 150 °C which is presumed to be approaching the highest temperature and shear boundary the lubricant may experience during engine operation (ASTM DS-62).

**Figure 3:**
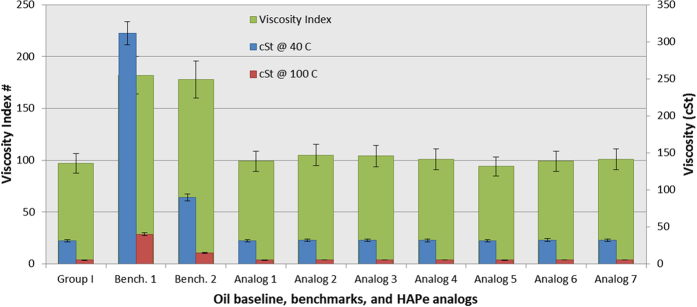
Viscosity data and index values as determined via a spindle viscometer. A standard 5% and 10% error was assigned to viscosity and viscosity index values, respectively.

For easy comparison of kinematic viscosity values and the respective VIs, of the benchmarks, base oil and the comb-burst analogs, the results are illustrated in the bar graph (Fig. 3). The ideal lubricant formulation would be homogeneous and undergo little viscosity change over a wide range of temperatures. Benchmark 1 and 2, in group I oil, are indeed homogeneous over a wide range of temperatures (10 to 100 °C). However, benchmark 1 significantly increased the viscosity of the neat oil from 31.4 cSt to 311.5 cSt at 40 °C. Furthermore, benchmark 1 demonstrated an undesirable increase in viscosity at 10 °C (1053 cSt) which may lead to problematic engine cold-starts, particularly in colder climates.

The viscosity index values reflect the overall resistance to the natural thinning tendency of lubricants and provide a unit-less number to compare one lubricant formulation to another. In general, a high viscosity index value with little effect on viscosity of the lubricant at lower temperatures is desirable. As calculated from data obtained from the spindle viscometer, the group I oil VI was 97 whereas benchmark 1 was 182 and benchmark 2 was 178 (2 and 1.67 wt %, respectively). The VI of group I oil suggests a more significant change in viscosity between 40 and 100 °C is observed as compared to the lubricant formulations with benchmark 1 and 2. The bar graph in Fig. 3 affords an illustrative comparison of the relationship between viscosity at 40 and 100 °C and their respective calculated VI values. The bar graph clearly indicates that benchmark 1 has a greater influence on viscosity whereas benchmark 2 has less of an impact on viscosity at 40 °C but still provides a comparable viscosity index value. In other words, benchmark 1 is inherently a better viscosity modifier (VM) whereas benchmark 2 is a viscosity index improver (VII). Significant towards the research described in this publication, benchmark 1 and 2 provide lubricant formulation models that encompass VM or VII behavior, which affords an opportunity to compare viscosity performance to our HAPe additives.

Lubricant formulations prepared with a 2 wt % concentration of our polymeric additives, HAPe, demonstrated little influence as a VM or VII. Little to no increase in viscosity was observed at 40 °C, which is desirable, while at 100 °C no increase in viscosity was observed with respect to the group I oil, which is undesirable. As a result, the VIs of the HAPe lubricant formulations were below those of the benchmarks. Furthermore, there was no clear connection between molecular weight and/or architecture, and viscosity results. For example, analog 2 (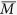
_*n*_ = 8.83 kg/mol) results suggested a small increase in viscosity from the neat group I oil at 40 and 100 °C (31.4 → 31.7 and 5.3 → 5.4 cSt, respectively) providing a slightly elevated VI of 105. Whereas analog 4 (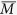
_*n*_ = 17.6 kg/mol) results indicate a slightly lower increase in viscosity (31.4 → 31.6 and 5.3 → 5.3 cSt, respectively) and VI (101). However, the changes in viscosity and respective VIs are within standard error and therefore do not provide insight into whether the suspected architectural differences may be influencing lubricant performance or not. In any case, the HAPe additives demonstrate little influence on the bulk oil’s viscosity.

Viscosity is a fluid’s ability to resist shear. For practical use, oils must meet the requirements set out in SAE engine oil viscosity classification J300^25^. This includes low and high temperature viscometry, as well as a high shear rate requirement of 1 × 10^6^ s^−1^ at 150 °C. High shear rate analysis under high temperature conditions provides insight into how a lubricant formulation may hold up in real world applications. The neat group I oil, benchmarks 1 and 2, and analog 4 viscosities were measured at 150 °C and a shear rate of 1 × 10^6^ s^−1^ to probe how these lubricant formulations would perform under limiting conditions. The viscosities of group I oil (2.3 cSt) and analog 4 (2.4 cSt) were ca. 44% of their respective viscosities at 100 °C. Benchmarks 1 (4.7 cSt) and 2 (2.6 cSt) demonstrated a more significant loss in viscosity under these conditions, respectively ca. 12% and 17% of their original viscosities at 100 °C. Although analog 4 has very little effect as a VI improver, the viscosity under these extreme conditions is actually higher than that of the oil. In this case, we can postulate that the retention of viscosity is due to the hyper-branched topology of the analog.

**Figure 4:**
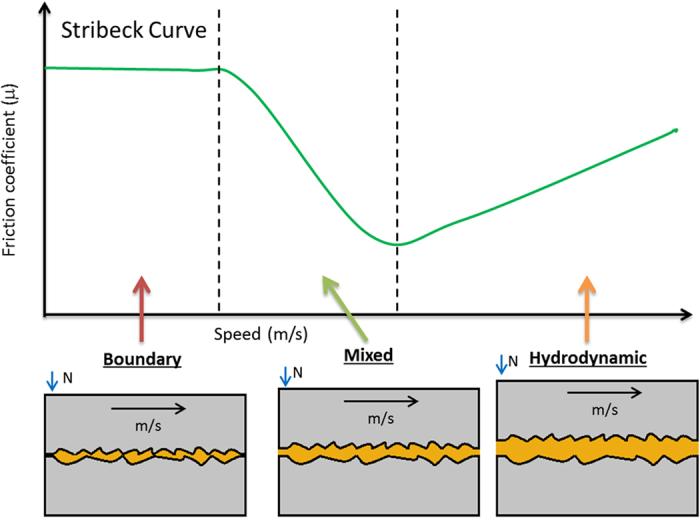
A Stribeck curve and illustrations of the 3 lubricant regimes occurring simultaneously in an engine.

Sliding friction in an engine is significantly reduced by the addition of lubricants. There are three regimes of lubrication, depending on the thickness of the lubricating film between the moving parts: boundary lubrication, mixed lubrication and hydrodynamic lubrication. In an operating engine, all three regimes occur simultaneously in different parts of the engine or under different conditions of operation (Fig. 4).

**Figure 5:**
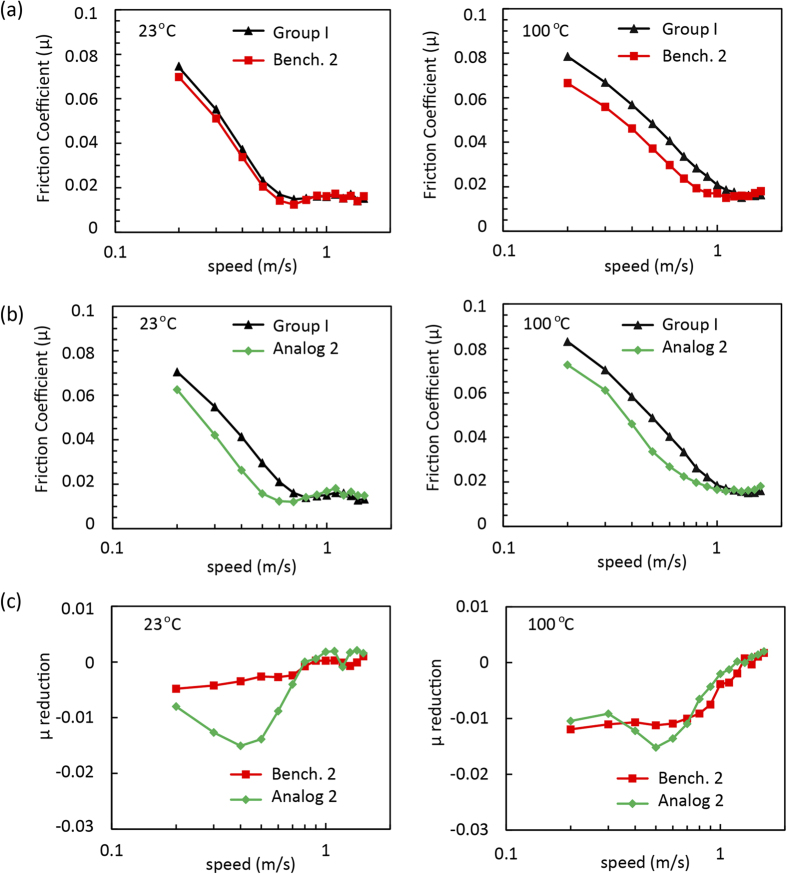
The Stribeck curves of (a) Bench. 2 (1.67 wt %) and (**b**) analog 2 (2 wt %) at 23 °C and 100 °C. (**c**) The reduction in friction coefficient achieved by subtracting the base oil friction.

Friction coefficients (μ) against speed (m/s) were measured at room temperature (~23 °C) and 100 °C on a variable-load journal bearing tester (VLBT), for lubricant formulations of benchmark 2 and analog 2. In Fig. 5, the friction trace of neat group I base oil was an average of two baseline tests performed before and after, each additized oil was tested.

In Fig. 5a, at 23 °C, benchmark 2 appears to have no influence on the friction versus the neat oil. At 100 °C, a reduction in μ was observed over the entire range. The gains in reducing friction at higher temperatures may be due to the benefit of a higher viscosity of benchmark 2 (15.1 cSt at 100 °C) versus the neat oil (5.3 cSt at 100 °C). In Fig. 5b, at both 23 and 100 °C, analog 2 shows a significant friction reduction in mixed and boundary lubrication at speeds <0.7 m/s. The observed reduction in friction may be attributed to a layer of polymeric additive deposited on the contact surfaces. This is not observed in the case of benchmark 2, where benchmark 2 has a more favorable viscosity than analog 2 (vide infra) and therefore should have a lower friction by default. It is conceivable that a thin polymeric film is formed at the interface, which lowers the coefficient of friction. The differences in viscosity between benchmark 2 (106 cSt at 23 °C; 15.1 cSt at 100 °C) and analog 2 (67 cSt at 23 °C; 5.4 cSt at 100 °C) make it hard to compare these two formulations directly. In Fig. 5c, the friction reduction of each additized oil was calculated by subtracting its friction trace from that of the base oil. At 23 °C, the more significant friction reduction in mixed and boundary lubrication regimes was produced by the lower-viscosity analog 2-additized oil compared with the benchmark 2-additized oil; at 100 °C, such reduction was only observed on the transition from boundary regime to mixed regime at ~0.5 m/s.

## Discussion

A comb-burst hyper-branched aryl polyester (HAPe) architecture was investigated as a lubricant additive. Four analogues were targeted with varying molecular weights and degrees of branching. The formation of an insoluble gel with polystyrene equivalent molecular weights (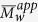) greater than ca. 60 kDa was readily observed while utilizing a previously published protocol where relatively higher molecular weights (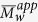 = 95 kDa) for their ethylene glycol analogue was reported^19^. This prompted us to modify the polycondensation reaction conditions by diluting the monomer in dichlorobenzene (DCB) affording greater control over the degree of polymerization while inherently reducing the degree of branching and promoting longer reaction times. Seven of these polymerizations were reported in the previous sections and include variations in the oligomeric linkage of the B component (AB_2_; 12 vs. 16 methylenes per monomer), post-functionalization groups (12 vs. 16 carbons per terminal alcohol), and 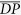 (6−26 repeating units). We envisioned the flexible core and corona of HAPe would afford an oil-soluble additive with viscosity index improvement results due to expected conformational changes and chain entanglement while reducing friction and shear degradation due to advantages afforded by non-linear architectures and incorporation of surface-interacting functional groups.

HAPes were blended homogeneously in group I oils above 50 °C with sufficient agitation and demonstrated modest improvements to the VI of the unadditized base oil. We observed little influence on the viscosity of the oil with 2 wt % HAPe at 40 °C (desirable). This trend was also observed at 100 °C (undesirable) indicating insufficient 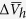 and chain entanglement. Due to the relationship of 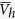 to 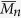, we believe this observation was caused by the relatively low 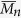
s of HAPes prepared in this study. Once again, 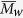
s were limited by solubility constraints, presumably caused by either the highly dense non-lipophilic components within the core (i.e. esters and aryls) or over entanglement leading to an insoluble HAPe network. The disadvantages of relatively low 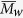
s and limited solubility proved to be more advantageous for friction reduction. At both 23 °C and 100 °C, a 2 wt % formulation of analog 2 (HAPe1 + C16; cSt = 67; 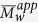 = 52.8 kDa) in group I base oil reduced friction more effectively than benchmark 2 (cSt = 106; 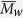 = ~200 kDa), suggesting great potential for analog 2 and similar materials as multi-functional lubricant additive.

Limitations in viscosity modification due to low molecular weights, may readily be overcome by utilizing other published syntheses of hyper-branched polymers that afford greater control via chain growth pathways^16^, non-linear architectures such as stars^26^, and highly branched polymers^9^. Further advances in preparing flexible, lipophilic hyper-branched polymers would also be advantageous for lubricant applications. Our ongoing interests include various non-linear architectures which may be exploited as a dual viscosity index improver and boundary friction modifier. These investigations will be published in the near future.

## Methods

### General considerations

Anhydrous solvents from septum sealed bottles were used as received to run reactions, while reagent grade solvents were utilized for transfers and purifications of products. Reactants and catalyst were used as received from suppliers. Triethylamine (TEA) was dried over activated molecular sieves (4 Å) prior to use. Anhydrous reaction glassware and equipment was oven dried and cooled under vacuum. These reaction vessels were backfilled with argon flowing through a tube filled with activated silica gel orange (drying agent). Thin layer chromatography (TLC; Baker-flex® precoated flexible sheets) was used as needed to monitor reactions. UV lamp and TLC staining agents (i.e. KMnO_4_) were employed to identify spots of interest. A group I oil (unadditized) from ExxonMobil was used as is to create a baseline data set for viscosity and friction measurements, clean in between measurements, and prepare weight concentrations of polymer in base oil. benchmarks 1 and 2 are commonly utilized lubricant viscosity modifiers employed here as comparative models and were kindly donated by industry partners.

### Characterization

Nuclear magnetic resonance spectroscopy (NMR) was utilized to confirm the chemical composition of monomers and polymers. Samples were dissolved in deuterated chloroform (CDCl_3_) containing tetramethylsilane (TMS, 0.3−1%, v/v) at which point the proton (^1^H) and carbon (^13^C) spectra were captured on a Varian 500 MHz instrument. TMS (δ = 0.00) and CDCl_3_ (δ = 7.26 (^1^H), 77.16 (^13^C)) were used as internal references for ^1^H NMR and ^13^C NMR respectively. Peaks were identified as singlets (s), doublets (d), triplets (t), multiplets (m), and broad (b). Integration was utilized to determine relative number of protons within the sample. Small molecule samples (i.e. monomers) were submitted for time of flight mass spectrometry (TOF-MS) analysis. Data is reported below. Polymers were submitted to Polymer Standard Service GmbH (PSS) for size exclusion chromatography (SEC) analysis which was conducted in THF and the molar mass as compared to polystyrene is reported.

### Lubricant investigations

Polymers were mixed into group I oil at concentrations of 1−3 wt %. Heat (50−120 °C) and agitation had to be employed to produce homogeneous blends. Several samples would become turbid and precipitate out at temperatures below 50 °C. This required additional agitation prior to viscosity and friction measurements. Dynamic viscosity was measured with several instruments, depending on the needs and temperature range of the instrument. The viscosity data was recorded in centipoise (cP) and converted into centistokes (cSt) by dividing the centipoise value by the density of the blend. The density (ρ = g/mL) of the neat oil and blends were measured at room temperature utilizing 5 mL volumetric flasks and a quantitative mass balance (4 decimal places). Three aliquots were measured for each oil blend at 2 wt % and these densities were averaged among themselves. We found that the densities of the additized oils were the same to the second decimal place (0.86 g/mL). The group I oil’s density was slightly lower (0.84 g/mL). A Brookfield viscometer was equipped with a cooling/heating jacket that was continuously flowing with oil supplied by an external cooling/heating bath that regulated the temperature at 40 and 100 °C. A rotating spindle (0.3−100 RPM) was submerged into the blended oil at the regulated temperatures and the dynamic shear was reported on the digital screen with a respective torsion percent. The cP value with the highest torsion percent was used for viscosity index calculations. Viscosity was measured by the dropping ball viscometer at 10 and 23 °C. A Tannas TBS viscometer was utilized to measure the viscosity of the blended oils at 150 °C while under a shear rate of 1 × 10^6^ s^−1^. A reference oil (R-350, 2.617 cP at 150 °C) was utilized to calibrate the instrument with a specific spindle height as well as provide a linear slope (RPM vs. shear rate) to interpolate the viscosities of the blended oils. A variable load-speed bearing tester (VLBT) was utilized to measure friction coefficients^28^. The load was applied through a stiff spring that was compressed by a ball screw at the end of the load arm. A 25.4 mm square coupon of A2 tool steel was used to press against a 25.4 mm diameter rotating steel bar of AISI 8620 alloy steel. A cartridge heater was installed under the coupon holder to control the temperature. The oil was supplied at the beginning of each test by filling the coupon holder. A 50 N normal load was applied for tests at 23 °C and a 26 N normal load was applied for those at 100 °C. The speed cycle was in a 0.1 m/s steps between 1.7 m/s and 0.2 m/s. The period for each step was 10 seconds. There were 3 cycles tested for each sample for a total of 480 seconds. The friction coefficients for the same speed from the 2^nd^ and the 3^rd^ cycle were averaged and plotted while 1^st^ cycle served as the running-in period with its data unused. Between each test, the tested oil was removed and the coupon and the bar were cleaned using isopropyl alcohol.

### Monomer Synthesis (AB_2_)^20^

#### Methyl 3,5-bis((8-hydroxyhexyl)oxy)benzoate (n = 4)

Methyl 3,5-dihydroxybenzoate (5.88 g, 0.035 mol), potassium carbonate (48.02 g, 0.35 mol), potassium iodide (~0.1 g), and 18-crown-6 (~0.025 g) were transferred into a 2-neck round bottom flask fitted with a condenser while under positive argon flow. The reaction flask was then degassed and left under vacuum for ca. 30 minutes. The reaction flask was backfilled with argon and left under an argon-inflated balloon. Acetonitrile (~300 mL) was transferred into the reaction flask via cannula and positive argon pressure. While stirring the mixture, 6-bromo-1-hexanol (13.94 g, 0.076 mol, ~2.2 equivalents) was added via syringe. The reaction was then heated until a medium reflux was achieved. In ca. 12 hours the reaction mixture turned brown and TLC analysis indicated the consumption of benzoate (5% MeOH/DCM; UV active). The insoluble species were removed by filtration, the filtrate was collected and the volatile organics were removed via roto-evaporation. The crude oil was re-dissolved in EtOAc (500 mL) and washed against 1 N HCl (500 mL) by agitating the biphasic layers in a separatory funnel. Fresh EtOAc (250 mL) and aqueous layer were agitated and separated three more times. The combined organic extracts were dried over anhydrous sodium sulfate, the solvent was removed, the residue dried and a viscous, amber color, oil was isolated (quantitatively) and analyzed by TLC, NMR, and MS. TLC (5% MeOH/DCM; UV): R_f_ = 0.30 (major), 0.32 (minor). ^1^H NMR (CDCl_3_, 500 MHz): δ 7.16 (s, 2 H), 6.63 (s, 1 H), 3.98 (t, 4 H, J = 5 Hz), 3.90 (s, 3 H), 3.66 (t, 4 H, J = 5 Hz), 1.80 (m, 4 H), 1.61 (m, 4 H), 1.55−1.36 (b, 10 H). ^13^C NMR (CDCl_3_, 125 MHz): δ 167.10, 160.21, 131.96, 107.78, 106.73, 68.27, 62.98, 52.34, 32.77, 29.24, 25.97, 25.63. HRMS (ESI): *m/z* 391.2079 (MNa^+^, [C_20_H_32_O_6_]Na^+^ requires 391.2097 g/mol).

#### Methyl 3,5-bis((8-hydroxyoctyl)oxy)benzoate (n = 6)

This monomer was prepared as described above with the exception of utilizing 8-bromo-1-octanol (~2.1 eq.). A yellow-orange viscous oil was isolated (quantitatively) and analyzed by TLC, NMR, and MS. TLC (5% MeOH/DCM; UV): R_f_ = 0.30 (major), 0.32 (minor). ^1^H NMR (CDCl_3_, 500 MHz): δ 7.16 (s, 2 H), 6.63 (s, 1 H), 3.97 (t, 4 H, J = 5 Hz), 3.90 (s, 3 H), 3.65 (t, 4 H, 5 Hz), 1.78 (m, 4 H), 1.58 (m, 4 H), 1.46 (m, 4 H), 1.41−1.29 (b, 14 H). ^13^C NMR (CDCl_3_, 125 MHz): δ 167.14, 160.23, 95.46, 68.37, 63.15, 52.35, 32.86, 29.42, 26.05, 25.78. HRMS (ESI): *m/z* 447.2724 (MNa^+^, [C_24_H_40_O_6_]Na^+^ requires 447.2723 g/mol).

### Polymerization Procedure^19^

#### Typical procedure

Without further purification, methyl 3,5-bis((8-hydroxyhexyl)oxy)benzoate (n = 4), obtained as described above, was dissolved in anhydrous dichlorobenzene (DCB) to a concentration of 1.0 M and kept under positive argon pressure. The reaction was fitted with a straight condenser and streaming N_2_ was utilized to continuously clear the head space. The reaction vessel was heated to 120 °C and then 1−3 drops of initiator, (*n*-Bu)_2_Sn(OAc)_2_, was added. The exterior temperature was then increased to 180 °C. Once TLC analysis indicated the majority of monomer was consumed, the reaction was subsequently followed by ^1^H NMR spectroscopy. Once the 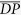 corresponded to a molecular weight of interest, the reaction mixture was removed from the heating mantle and allowed to cool to room temperature. Bubbling nitrogen through the crude solution removed the majority of DCB. The remaining viscous oil was diluted in DCM and precipitated into cold Et_2_O (−40 °C). The precipitate isolated from vacuum filtration was collected and placed under high vacuum for ca. 16 hours, to obtain an amber viscous oil in yields greater than 50%.

**HAPe1** (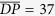, 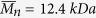). ^1^H NMR (CDCl_3_, 500 MHz): δ 7.15 (s, 2.11 H), 6.62 (s, 1.00 H), 4.30 (s, 2.06 H), 3.97 (s, 3.61 H), 3.89 (s, 0.14 H), 3.65 (s, 1.48 H), 1.79 (s, 7.39 H), 1.71−1.31 (b, 11.18 H).

**HAPe2** (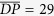, 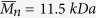). ^1^H NMR (CDCl_3_, 500 MHz): δ 7.14 (s, 2.01 H), 6.61 (s, 1.00 H), 4.28 (t, 1.96 H, J = 5 Hz), 3.95 (t, 4.09 H, J = 5 Hz), 3.88 (s, 0.10 H), 3.63 (b, 2.35 H), 1.81−1.68 (b, 7.10 H), 1.61−1.50 (b, 3.16 H), 1.50−1.40 (b, 7.08 H), 1.40−1.29 (b, 11.84).

### Post-modification of HAPes^21^

#### Typical Procedure

HAPe1 (~3.89 g; 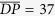, 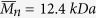) was dissolved in anhydrous THF (20 mL) and transferred into a 2-neck reaction flask. The flask was placed into an ice bath (0 °C) for ca. 15 minutes. While under an argon balloon, Et_3_N (ca. 2 eq.) was injected into the flask. Dodecanoyl chloride (C12, ca. 2 eq.) was added slowly to the mixture via syringe. The reaction was allowed to warm to room temperature and stirred overnight. The resulting ammonium salt was removed by vacuum filtration and rinsed with THF. The filtrate and wash were collected, combined and concentrated to 10 mL volume via roto-evaporation. MeOH (~5 mL) was added to the crude mixture to quench remaining acyl chlorides and stirred for 20 minutes, followed by solvent removal. The resulting waxy material was re-dissolved in DCM and the concentrated polymer solution was precipitated into cold Et_2_O (−40 °C). The precipitate was collected via vacuum filtration. This purification was repeated once more and the resulting residue dried under vacuum for ca. 16 hours. A viscous amber oil was collected with a typical gravimetric yield of ca. 50%.

**HAPe1 + C12** (**1**; 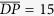, 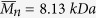). ^1^H NMR (CDCl_3_, 500 MHz): δ 7.14 (s, 2.05 H), 6.61 (s, 1.00 H), 4.29 (b, 1.88 H), 4.06 (b, 2.53 H), 3.95 (b, 4.55 H), 3.88 (s, 0.21 H), 2.27 (m, 1.41 H), 1.7−1.55 (b, 5.27 H), 1.55−1.36 (b, 10.80 H), 1.34−1.15 (b, 14.70 H), 0.86 (b, 2.62 H). SEC (PS cal.): 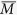
_*n*_^app^ = 17.1 kg/mol, 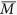
_*w*_^app^ = 55.7 kg/mol, 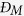 = 3.3, multimodal.

**HAPe1 + C16** (**2**; 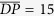, 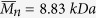). ^1^H NMR (CDCl_3_, 500 MHz): δ 7.14 (s, 2.02 H), 6.60 (s, 1.00 H), 4.29 (b, 2.06 H), 4.05 (b, 2.55 H), 3.95 (b, 4.32 H), 3.87 (s, 0.20 H), 2.27 (b, 1.48 H), 1.78 (b, 7.41), 1.71−1.55 (b, 5.89 H), 1.55−1.44 (b, 7.55 H), 1.44−1.37 (b, 3.84 H), 1.32−1.17 (b, 24.25 H), 0.85 (b, 2.68 H). ^13^C NMR (CDCl_3_, 125 MHz): δ 174.01, 171.23, 166.50, 160.08, 132.19, 107.70, 106.20, 70.85, 68.10, 65.12, 64.22, 34.41, 31.98, 29.74, 29.21, 28.70, 25.84, 25.06, 22.76, 21.06, 14.21. SEC (PS cal.): 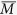
_*n*_^app^ = 18.2 kg/mol, 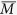
_*w*_^app^ = 52.8 kg/mol, 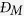 = 2.9, multimodal.

**HAPe2 + C12** (**3**; 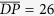, 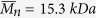). ^1^H NMR (CDCl_3_, 500 MHz): δ 7.14 (s, 2.16 H), 6.61 (s, 1.00 H), 4.28 (t, 1.95 H, J = 5 Hz), 4.05 (t, 2.40 H, J = 5 Hz), 3.95 (m, 5.46 H), 3.88 (s, 0.12 H), 2.28 (m, 2.25 H), 1.77 (m, 8.88 H), 1.61 (s, 5.58), 1.52−1.16 (m, 78.86 H), 0.87 (m, 10.23 H). ^13^C NMR (CDCl_3_, 125 MHz): δ 174.17, 169.97, 166.67, 160.19, 145.67, 132.30, 107.73, 106.25, 101.88, 68.34, 65.34, 64.46, 53.84, 34.53, 32.04, 31.09, 29.73, 29.45, 28.78, 27.63, 26.47, 26.09, 25.15, 24.77, 22.82, 14.27. SEC (PS cal.): 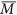
_*n*_^app^ = 14.7 kg/mol, 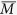
_*w*_^app^ = 25.9 kg/mol, 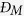 = 1.7, multimodal.

**HAPe2 + C16** (**4**; 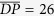, 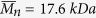). ^1^H NMR (CDCl_3_, 500 MHz): δ 7.14 (s, 2.31 H), 6.61 (s, 1.00 H), 4.28 (t, 2.10 H, J = 5 Hz), 4.05 (t, 2.78 H, J = 5 Hz), 3.95 (m, 6.25 H), 3.88 (s, 0.13 H), 2.29 (m, 3.27 H), 1.76 (s, 11.07), 1.61 (s, 9.73 H), 1.52−1.13 (m, 156.60 H), 0.87 (t, 12.62 H, J = 5 Hz). ^13^C NMR (CDCl_3_, 125 MHz): δ 174.50, 169.94, 166.65, 160.18, 145.67, 132.29, 107.72, 106.23, 101.85, 68.32, 65.33, 64.44, 53.83, 51.57, 34.51, 34.24, 32.06, 29.80, 29.61, 29.50, 29.40, 29.32, 28.77, 26.46, 26.08, 25.14, 24.76, 22.83, 14.26. SEC (PS cal.): 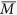
_*n*_^app^ = 15.1 kg/mol, 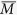
_*w*_^app^ = 25.7 kg/mol, 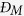 = 1.7, multimodal.

## Additional Information

**How to cite this article**: Robinson, J. W. *et al.* Probing the molecular design of hyper-branched aryl polyesters towards lubricant applications. *Sci. Rep.* **6**, 18624; doi: 10.1038/srep18624 (2016).

## Supplementary Material

Supplementary Information

## Figures and Tables

**Figure 1:**
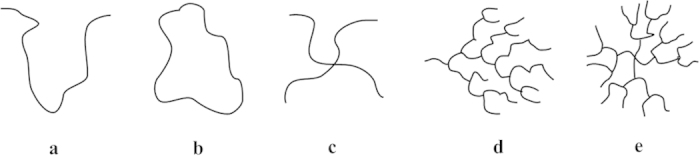
Traditional cartoons of polymers with the following architectures (**a**) Linear, (**b**) Ring, (**c**) Star, (**d**) Hyper-branched, and (**e**) Dendrimer.

**Table 1:** Characterization of HAPes.

Analog	Composition^a^	^1^H NMR^b^				SEC^d^		
		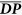 c	P_f_%^c^	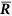 c	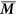 _*n*_ (kg/mol)^c^	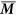 _*n*_^app^ (kg/mol)	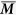 _*w*_^app^ (kg/mol)	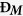
1	HAPe1 + C12	16	98	17	8.3 5.2 + (3.1)	17.1	55.7	3.3
2	HAPe1 + C16	16	98	19	9.86 5.36 + (4.5)	18.2	52.8	2.9
3	HAPe2 + C12	26	89	28	15.3 10.2 + (5.1)	14.7	25.9	1.7
4	HAPe2 + C16	26	66	31	17.6 10.2 + (7.4)	15.1	25.7	1.7
5	HAPe2 + C16	6	49	3	3.05 2.35 + (0.7)	4.75	7.35	1.6
6	HAPe2 + C16	46	>99	50	29.9 18.0 + (11.9)	6.1	17	2.8
7	HAPe2 + C16	<56	96	53	34.5 21.9 + (12.6)	9.86	26.5	2.7

^a^HAPe1 (n = 4) and HAPe2 (n = 6) were subjected to post-modification with palmitoyl chloride (C16) or dodecanoyl chloride (C12).

^b^Nuclear magnetic resonance (NMR) spectrometry with tetramethylsilane (TMS, δ = 0.00) as an internal reference for proton (^1^H) analysis in deuterated chloroform (CDCl_3_).

^c^see supporting information for details on calculations of average degree of polymerization (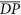), percent of post-functionalization (P_f_%), average number of peripheral groups (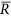), and number-average molecular weight (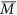
_*n*_).

^d^apparent number-average molecular weight (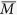
_*n*_^app^) and molar dispersity (Đ_M_) were determined by utilizing polystyrene standards via size exclusion chromatography (SEC) analysis.

**Table 2:** Determined kinematic viscosity (cSt) values and respective viscosity indices (VIs) at 2 wt %.

Analog	Dropping Ball Viscometer (cSt)^d^		Spindle Viscometer (cSt)^d^		
	10 °C	23 °C	40 °C	100 °C	VI
Group I^a^	n.d.^e^	n.d.	31.4	5.3	97
Bench. 1^b^	1053	430	311.5	40.1	182
Bench. 2^c^	229	106	89.8	15.1	178
1	152	67	31.5	5.3	99
2	153	67	31.7	5.4	105
3	n.d.	n.d.	31.9	5.4	104
4	n.d.	n.d.	31.6	5.3	101
5	n.d.	n.d.	30.9	5.2	94
6	157	71	32.4	5.4	99
7	156	69	32.1	5.4	101

^a^Group I oil density = 0.84 g/cm^3^.

^b^VII additized (2 wt % polymer) group I oil density = 0.86 g/cm^3^.

^c^benchmark (Bench.) 2 corrected wt % is 1.67.

^d^
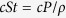

^e^not determined (n.d.).

## References

[b1] Rudnick L. R. Lubricant Additives: Chemistry and Applications. 2nd (ed. Rudnick L. R. ) pp 777 (CRC Press, 2009).

[b2] Tamoto Y., Kido M. & Murata H. Possibilities of ultra low viscosity fuel saving gasoline engine oil. *Soc. Automot. Eng*. SP-1885, 143–148 (2004).

[b3] Covitch M. J. *et al.* Extending SAE J300 to Viscosity Grades below SAE 20. SAE International Journal of Fuels and Lubricants. 3, 1030–1040 (2010).

[b4] Khemchandani B. & Verma H. S. High performance shear stable viscosity modifiers. Adv. Mater. Sci. 1, 33–41 (2013).

[b5] Rizvi S. Q. Lubricant Chemistry, Technology, Selection, and Design. pp 657 (ASTM International, 2009).

[b6] Stöhr T., Eisenberg B. & Müller M. A new generation of high performance viscosity modifiers based on comb polymers. SAE Int. J. Fuels Lubr. 1, 1511–1516 (2008).

[b7] Visger D., Davies M., Price D., Baum M. & Schober B. The Lubrizol Corporation, assignee. Star Polymers and Compositions Thereof. United States Patent US 20,070,244,018. 2007 October 18.

[b8] Brzytwa A. J. & Johnston J. Scaled production of RAFT CTA—a star performer. Polym. Prepr., Am. Chem. Soc., Div. Polym. Chem. 52, 533–534 (2011).

[b9] Wang J. L., Ye Z. B. & Zhu S. P. Topology-engineered hyperbranched high-molecular-weight polyethylenes as lubricant viscosity-index improvers of high shear stability. Ind. Eng. Chem. Res. 46, 1174–1178 (2007).

[b10] Lu Y., An L. & Wang Z.-G. Intrinsic Viscosity of Polymers: General Theory Based on a Partially Permeable Sphere Model. Macromolecules. 46, 5731–5740 (2013).

[b11] Sendijarevic I. & McHugh A. J. Effects of molecular variables and architecture on the rheological behavior of dendritic polymers. Macromolecules. 33, 590–596 (2000).

[b12] Frechet J. M. J. Functional Polymers and Dendrimers—Reactivity, Molecular Architecture, and Interfacial Energy. Science. 263, 1710–1715 (1994). 8134834 10.1126/science.8134834

[b13] Hawker C. J., Farrington P. J., Mackay M. E., Wooley K. L. & Frechet J. M. J. Molecular Ball-Bearings—the Unusual Melt Viscosity Behavior of Dendritic Macromolecules. J. Am. Chem. Soc. 117, 4409–4410 (1995).

[b14] Covitch M. J. & Trickett K. J. How Polymers Behave as Viscosity Index Improvers in Lubricating Oils. Adv. Chem. Eng. Sci. 5, 134 (2015).

[b15] Zagar E. & Zigon M. Aliphatic hyperbranched polyesters based on 2,2-bis(methylol)propionic acid-Determination of structure, solution and bulk properties. Prog. Polym. Sci. 36, 53–88 (2011).

[b16] Voit B. I. & Lederer A. Hyperbranched and Highly Branched Polymer Architectures-Synthetic Strategies and Major Characterization Aspects. Chem. Rev. 109, 5924–5973 (2009). 19785454 10.1021/cr900068q

[b17] Lach C., Muller P., Frey H. & Mulhaupt R. Hyperbranched polycarbosilane macromonomers bearing oxazoline functionalities. Macromol. Rapid Comm. 18, 253–260 (1997).

[b18] Gao C. & Yan D. Hyperbranched polymers: from synthesis to applications. Prog. Polym. Sci. 29, 183–275 (2004).

[b19] Hawker C. J., Chu F. K., Pomery P. J. & Hill D. J. T. Hyperbranched poly(ethylene glycol)s: A new class of ion-conducting materials. Macromolecules. 29, 3831–3838 (1996).

[b20] Saha A. & Ramakrishnan S. AB(2) plus A type copolymerization approach for the preparation of thermosensitive PEGylated hyperbranched polymers. Macromolecules. 41, 5658–5664 (2008).

[b21] Sunder A., Krämer M., Hanselmann R., Mülhaupt R. & Frey H. Molecular nanocapsules based on amphiphilic hyperbranched polyglycerols. Angew. Chem. Int. Ed. 38, 3552–3555 (1999). 10.1002/(sici)1521-3773(19991203)38:23<3552::aid-anie3552>3.3.co;2-7 10602240

[b22] Wooley K. L., Hawker C. J., Lee R. & Frechet J. M. J. One-Step Synthesis of Hyperbranched Polyesters—Molecular-Weight Control and Chain-End Functionalization. Polym. J. 26, 187–197 (1994).

[b23] Shu C. F. & Leu C. M. Hyperbranched poly(ether ketone) with carboxylic acid terminal groups: Synthesis, characterization, and derivatization. Macromolecules. 32, 100–105 (1999).

[b24] Uniteasy, Calculation of Viscosity Index. (2008) Available at: www.uniteasy.com/en/unitsCon/calvi.htm. (Accessed: 23^rd^ September 2015).

[b25] SAE International, Engine Oil Viscosity Classification. SAE Standard J300-201501. (2015) Available at: http://standards.sae.org/j300_201501/ (Accessed: 23^rd^ September 2015).

[b26] Moad G., Rizzardo E. & Thang S. H. RAFT Polymerization and Some of its Applications. Chem-Asian J. 8, 1634–1644 (2013). 23606667 10.1002/asia.201300262

