# Supplementary material for: Probing the molecular design of hyper-branched aryl polyesters towards lubricant applications
Source: Sci Rep. 2016 Jan 5;6:18624. doi: 10.1038/srep18624 (PMC4700471; doi:10.1038/srep18624)
Supplement: Supplementary Information [file srep18624-s1.pdf]

Probing the molecular design of hyper-branched aryl polyesters towards lubricant applications

Joshua W. Robinson[1], Yan Zhou[2], Priyanka Bhattacharya[1], Robert Erck[3], Jun Qu[2], J. Timothy Bays[1], Lelia Cosimbescu[1,*]

[1]Pacific Northwest National Laboratory, Richland, Washington 99352
[2]Oak Ridge National Laboratory, Oak Ridge, Tennessee 37831
[3]Argonne National Laboratory, Lemont, Illinois 60439
*lelia.cosimbescu@pnnl.gov

Supporting information

General considerations. Anhydrous solvents from septum sealed bottles were used as received to run reactions, while reagent grade solvents were utilized for transfers and purifications of products. Reactants and catalyst were used as received from suppliers. Triethylamine (TEA) was dried over activated molecular sieves (4 Å) prior to use. Anhydrous reaction glassware and equipment was oven dried and cooled under vacuum. These reaction vessels were backfilled with argon flowing through a tube filled with activated silica gel orange (drying agent). Thin layer chromatography (TLC; Baker-flex® precoated flexible sheets) was used as needed to monitor reactions. UV lamp and TLC staining agents (i.e. $KMnO_4$) were employed to identify spots of interest. A group I oil (unadditized) from ExxonMobil was used as is to create a baseline data set for viscosity and friction measurements, clean in between measurements, and prepare weight concentrations of polymer in base oil. Bench.s 1 and 2 are commonly utilized lubricant viscosity modifiers employed here as comparative models and were kindly donated by industry partners.

Characterization. Nuclear magnetic resonance spectroscopy (NMR) was utilized to confirm the chemical composition of monomers and polymers. Samples were dissolved in deuterated chloroform ($CDCl_3$) containing tetramethylsilane (TMS, 0.3–1 %, v/v) at which point the proton (^{1}H) and carbon (^{13}C) spectra were captured on a Varian 500 MHz instrument. TMS (δ = 0.00) and $CDCl_3$ (δ = 7.26 (^{1}H), 77.16 (^{13}C)) were used as internal references for ^{1}H NMR and ^{13}C NMR respectively. Peaks were identified as singlets (s), doublets (d), triplets (t), multiplets (m), and broad (b). Integration was utilized to determine relative number of protons within the sample. Small molecule samples (i.e. monomers) were submitted for time of flight mass spectrometry (TOF-MS) analysis. Data is reported below. Polymers were submitted to Polymer Standard Service GmbH (PSS) for size exclusion chromatography (SEC) analysis which was conducted in THF and the molar mass as compared to polystyrene is reported.

*Lubricant investigations***.** Polymers were mixed into group I oil at concentrations of 1–3 wt %. Heat (50–120 °C) and agitation had to be employed to produce homogeneous blends. Several samples would become turbid and precipitate out at temperatures below 50 °C. This required

additional agitation prior to viscosity and friction measurements. Dynamic viscosity was measured with several instruments, depending on the needs and temperature and shear range of the instrument. The viscosity data was recorded in centipoise (cP) and converted into centistokes (cSt) by dividing the centipoise value by the density of the blend. A Brookfield viscometer was equipped with a cooling/heating jacket that was continuously flowing with oil supplied by an external cooling/heating bath that regulated the temperature at 40 and 100 °C. A rotating spindle (0.3–100 RPM) was submerged into the blended oil at the regulated temperatures and the dynamic shear was reported on the digital screen with a respective torsion percent. The cP value with the highest torsion percent was used for viscosity index calculations. Viscosity was measured by the dropping ball viscometer at 10 and 23 °C. A Tannas TBS viscometer was utilized to measure the viscosity of the blended oils at 150 °C while under a shear rate of 1x10^6 s^{-1}. A reference oil (R-350, 2.617 cP at 150 °C) was utilized to calibrate the instrument with a specific spindle height as well as provide a linear slope (RPM vs. shear rate) to interpolate the viscosities of the blended oils. A variable load-speed bearing tester (VLBT) was utilized to measure friction coefficients.[20] The load was applied through a stiff spring that was compressed by a ball screw at the end of the load arm. A 25.4 mm square coupon of A2 tool steel was used to press against a 25.4 mm diameter rotating steel bar of AISI 8620 alloy steel. A cartridge heater was installed under the coupon holder to control the temperature. The oil was supplied at the beginning of each test by filling the coupon holder. A 50 N normal load was applied for tests at 23 °C and a 26 N normal load was applied for those at 100 °C. The speed cycle was in a 0.1 m/s steps between 1.7 m/s and 0.2 m/s. The period for each step was 10 seconds. There were 3 cycles tested for each sample for a total of 480 seconds. The friction coefficients for the same speed from the 2nd and the 3rd cycle were averaged and plotted while 1st cycle served as the running-in period with its data unused. Between each test, the tested oil was removed and the coupon and the bar were cleaned using isopropyl alcohol.

Monomer Synthesis (AB_2).[21]

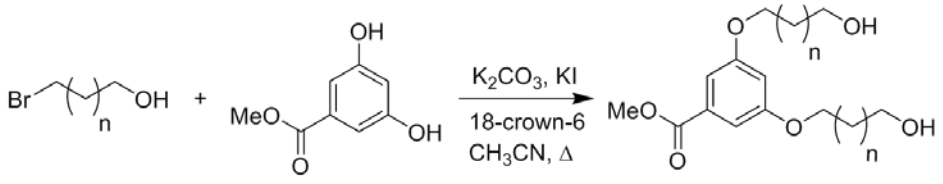

n = 4 or 6

S1. Monomer Synthesis

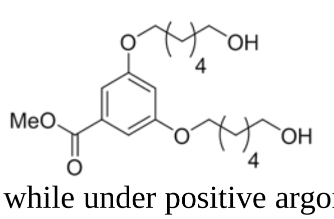

Methyl 3,5-bis((8-hydroxyhexyl)oxy)benzoate (n = 4). Methyl 3,5-dihydroxybenzoate (5.88 g, 0.035 mol), potassium carbonate (48.02 g, 0.35 mol), potassium iodide (~0.1 g), and 18-crown-6 (~0.025 g) were transferred into a 2-neck round bottom flask fitted with a condenser while under positive argon flow. The reaction flask was then degassed and left under vacuum for

ca. 30 minutes. The reaction flask was backfilled with argon and left under an argon-inflated balloon. Acetonitrile (~300 mL) was transferred into the reaction flask via cannula and positive argon pressure. While stirring the mixture, 6-bromo-1-hexanol (13.94 g, 0.076 mol, ~2.2 equivalents) was added via syringe. The reaction was then heated until a medium reflux was achieved. In ca. 12 hours the reaction mixture turned brown and TLC analysis indicated the consumption of benzoate (5% MeOH/DCM; UV active). The insoluble species were removed by filtration, the filtrate was collected and the volatile organics were removed via roto-evaporation. The crude oil was re-dissolved in EtOAc (500 mL) and washed against 1 N HCl (500 mL) by agitating the biphasic layers in a separatory funnel. Fresh EtOAc (250 mL) and aqueous layer were agitated and separated three more times. The combined organic extracts were dried over anhydrous sodium sulfate, the solvent was removed , the residue dried and a viscous, amber color, oil was isolated (quantitatively) and analyzed by TLC, NMR, and MS. TLC (5% MeOH/DCM; UV): R_f = 0.30 (major), 0.32 (minor). ^{1}H NMR ($CDCl_3$, 500 MHz): δ 7.16 (s, 2H), 6.63 (s, 1H), 3.98 (t, 4H, J = 5 Hz), 3.90 (s, 3H), 3.66 (t, 4H, J = 5 Hz), 1.80 (m, 4H), 1.61 (m, 4H), 1.55–1.36 (b, 10 H). ^{13}C NMR ($CDCl_3$, 125 MHz): δ 167.10, 160.21, 131.96, 107.78, 106.73, 68.27, 62.98, 52.34, 32.77, 29.24, 25.97, 25.63. HRMS (ESI): *m/z* 391.2079 (MNa^+, $[C_{20}H_{32}O_6]Na^+$ requires 391.2097 g/mol).

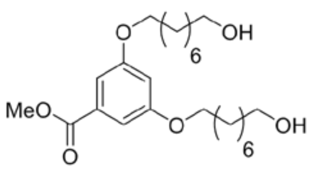

Methyl 3,5-bis((8-hydroxyoctyl)oxy)benzoate (n = 6). This monomer was prepared as described above with the exception of utilizing 8-bromo-1-octanol (~2.1 eq.). A yellow-orange viscous oil was isolated (quantitatively) and analyzed by TLC, NMR, and MS. TLC (5% MeOH/DCM; UV): R_f = 0.30 (major), 0.32 (minor). ^{1}H NMR ($CDCl_3$, 500 MHz): δ 7.16 (s, 2H), 6.63 (s, 1H), 3.97 (t, 4H, J = 5 Hz), 3.90 (s, 3H), 3.65 (t, 4H, 5 Hz), 1.78 (m, 4H), 1.58 (m, 4H), 1.46 (m, 4H), 1.41–1.29 (b, 14H). ^{13}C NMR ($CDCl_3$, 125 MHz): δ 167.14, 160.23, 95.46, 68.37, 63.15, 52.35, 32.86, 29.42, 26.05, 25.78. HRMS (ESI): *m/z* 447.2724 (MNa^+, $[C_{24}H_{40}O_6]Na^+$ requires 447.2723 g/mol).

Polymerization Procedure.[19]

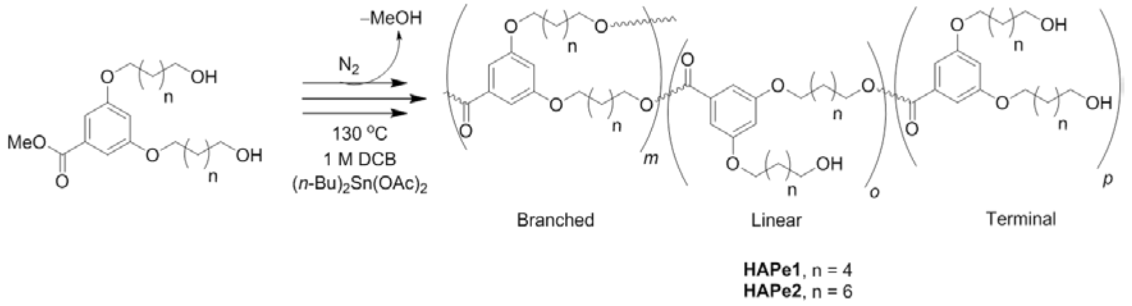

S2. Polymer Synthesis

Typical procedure: Without further purification, methyl 3,5-bis((8-hydroxyhexyl)oxy)benzoate (n = 4), obtained as described above, was dissolved in anhydrous dichlorobenzene (DCB) to a

concentration of 1.0 M and kept under positive argon pressure. The reaction was fitted with a straight condenser and streaming N_2 was utilized to continuously clear the head space. The reaction vessel was heated to 120 °C and then 1–3 drops of initiator, $(n\text{-Bu})_2Sn(OAc)_2$, was added. The exterior temperature was then increased to 180 °C. Once TLC analysis indicated the majority of monomer was consumed, the reaction was subsequently followed by ^{1}H NMR spectroscopy. Once the $\overline{DP}$ corresponded to a molecular weight of interest, the reaction mixture was removed from the heating mantle and allowed to cool to room temperature. Bubbling nitrogen through the crude solution removed the majority of DCB. The remaining viscous oil was diluted in DCM and precipitated into cold Et_2O (–40 °C). The precipitate isolated from vacuum filtration was collected and placed under high vacuum for ca. 16 hours, to obtain an amber viscous oil in yields greater than 50%.

HAPe1 ($\overline{DP} = 37,\ \bar{M}_n = 12.4\ kDa$). ^{1}H NMR ($CDCl_3$, 500 MHz): δ 7.15 (s, 2.11H), 6.62 (s, 1.00H), 4.30 (s, 2.06H), 3.97 (s, 3.61H), 3.89 (s, 0.14H), 3.65 (s, 1.48H), 1.79 (s, 7.39H), 1.71–1.31 (b, 11.18H).

HAPe2 ($\overline{DP} = 29,\ \bar{M}_n = 11.5\ kDa$). ^{1}H NMR ($CDCl_3$, 500 MHz): δ 7.14 (s, 2.01H), 6.61 (s, 1.00H), 4.28 (t, 1.96H, J = 5 Hz), 3.95 (t, 4.09H, J = 5 Hz), 3.88 (s, 0.10H), 3.63 (b, 2.35H), 1.81–1.68 (b, 7.10H), 1.61–1.50 (b, 3.16H), 1.50–1.40 (b, 7.08H), 1.40–1.29 (b, 11.84).

Post-modification of HAPes.[22]

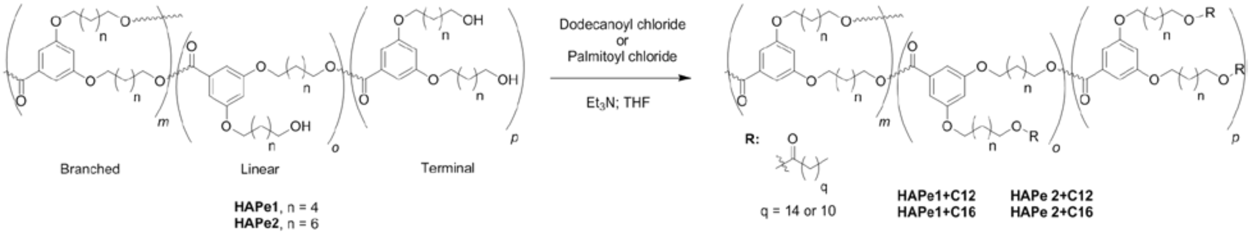

S3. Polymer post-modification

Typical Procedure: HAPe1 (~3.89 g; $\overline{DP} = 37,\ \bar{M}_n = 12.4\ kDa$) was dissolved in anhydrous THF (20 mL) and transferred into a 2-neck reaction flask. The flask was placed into an ice bath (0 °C) for ca. 15 minutes. While under an argon balloon, Et_3N (ca. 2 eq.) was injected into the flask. Dodecanoyl chloride (C12, ca. 2 eq.) was added slowly to the mixture via syringe. The reaction was allowed to warm to room temperature and stirred overnight. The resulting ammonium salt was removed by vacuum filtration and rinsed with THF. The filtrate and wash were collected, combined and concentrated to 10 mL volume via roto-evaporation. MeOH (~5 mL) was added to the crude mixture to quench remaining acyl chlorides and stirred for 20 minutes, followed by solvent removal. The resulting waxy material was re-dissolved in DCM and the concentrated polymer solution was precipitated into cold Et_2O (–40 °C). The precipitate was collected via vacuum filtration. This purification was repeated once more and the resulting

residue dried under vacuum for ca. 16 hours. A viscous amber oil was collected with a typical gravimetric yield of ca. 50%.

HAPe1+C12 (**1**; $\overline{DP} = 15$, $\bar{M}_n = 8.13\ kDa$). ^{1}H NMR ($CDCl_3$, 500 MHz): δ 7.14 (s, 2.05H), 6.61 (s, 1.00H), 4.29 (b, 1.88H), 4.06 (b, 2.53H), 3.95 (b, 4.55H), 3.88 (s, 0.21H), 2.27 (m, 1.41H), 1.7–1.55 (b, 5.27H), 1.55–1.36 (b, 10.80H), 1.34–1.15 (b, 14.70H), 0.86 (b, 2.62H). SEC (PS cal.): $\bar{M}_n^{app}$ = 17.1 kg/mol, $\bar{M}_w^{app}$ = 55.7 kg/mol, $Đ_M$ = 3.3, multimodal.

HAPe1+C16 (**2**; $\overline{DP} = 15$, $\bar{M}_n = 8.83\ kDa$). ^{1}H NMR ($CDCl_3$, 500 MHz): δ 7.14 (s, 2.02H), 6.60 (s, 1.00H), 4.29 (b, 2.06H), 4.05 (b, 2.55H), 3.95 (b, 4.32H), 3.87 (s, 0.20H), 2.27 (b, 1.48H), 1.78 (b, 7.41), 1.71–1.55 (b, 5.89H), 1.55–1.44 (b, 7.55H), 1.44–1.37 (b, 3.84H), 1.32–1.17 (b, 24.25H), 0.85 (b, 2.68H). ^{13}C NMR ($CDCl_3$, 125 MHz): δ 174.01, 171.23, 166.50, 160.08, 132.19, 107.70, 106.20, 70.85, 68.10, 65.12, 64.22, 34.41, 31.98, 29.74, 29.21, 28.70, 25.84, 25.06, 22.76, 21.06, 14.21. SEC (PS cal.): $\bar{M}_n^{app}$ = 18.2 kg/mol, $\bar{M}_w^{app}$ = 52.8 kg/mol, $Đ_M$ = 2.9, multimodal.

HAPe2+C12 (**3**; $\overline{DP} = 26$, $\bar{M}_n = 15.3\ kDa$). ^{1}H NMR ($CDCl_3$, 500 MHz): δ 7.14 (s, 2.16H), 6.61 (s, 1.00H), 4.28 (t, 1.95H, J= 5 Hz), 4.05 (t, 2.40H, J = 5 Hz), 3.95 (m, 5.46H), 3.88 (s, 0.12H), 2.28 (m, 2.25H), 1.77 (m, 8.88H), 1.61 (s, 5.58), 1.52–1.16 (m, 78.86H), 0.87 (m, 10.23H). ^{13}C NMR ($CDCl_3$, 125 MHz): δ 174.17, 169.97, 166.67, 160.19, 145.67, 132.30, 107.73, 106.25, 101.88, 68.34, 65.34, 64.46, 53.84, 34.53, 32.04, 31.09, 29.73, 29.45, 28.78, 27.63, 26.47, 26.09, 25.15, 24.77, 22.82, 14.27. SEC (PS cal.): $\bar{M}_n^{app}$ = 14.7 kg/mol, $\bar{M}_w^{app}$ = 25.9 kg/mol, $Đ_M$ = 1.7, multimodal.

HAPe2+C16 (**4**; $\overline{DP} = 26$, $\bar{M}_n = 17.6\ kDa$). ^{1}H NMR ($CDCl_3$, 500 MHz): δ 7.14 (s, 2.31H), 6.61 (s, 1.00H), 4.28 (t, 2.10H, J = 5 Hz), 4.05 (t, 2.78H, J = 5 Hz), 3.95 (m, 6.25H), 3.88 (s, 0.13H), 2.29 (m, 3.27H), 1.76 (s, 11.07), 1.61 (s, 9.73H), 1.52–1.13 (m, 156.60H), 0.87 (t, 12.62H, J = 5 Hz). ^{13}C NMR ($CDCl_3$, 125 MHz): δ 174.50, 169.94, 166.65, 160.18, 145.67, 132.29, 107.72, 106.23, 101.85, 68.32, 65.33, 64.44, 53.83, 51.57, 34.51, 34.24, 32.06, 29.80, 29.61, 29.50, 29.40, 29.32, 28.77, 26.46, 26.08, 25.14, 24.76, 22.83, 14.26. SEC (PS cal.): $\bar{M}_n^{app}$ = 15.1 kg/mol, $\bar{M}_w^{app}$ = 25.7 kg/mol, $Đ_M$ = 1.7, multimodal.

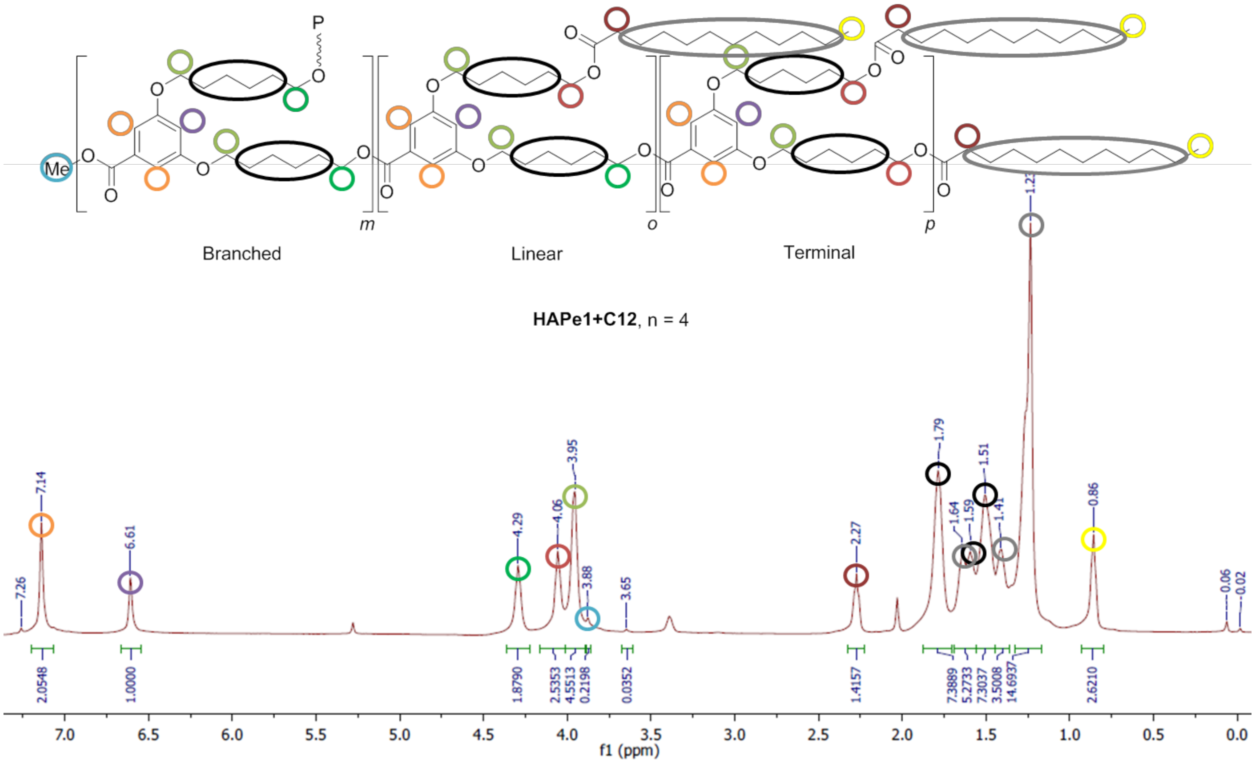

S4. ^{1}HNMR spectrum of post-modified HAPe with peak assignments

The average degree of polymerization ($\overline{DP} = \bar{m} + \bar{o} + \bar{p}$) was calculated using the integration of the protons associated with the methyl ester (blue circle, □ = 3.88; and the methylene peaks of the alkyl aryl ether (lime circles, δ = 3.95; 4.5513H/4H = 1.1378; 1.1378/0.07326 = 15.5). The number average molecular weight ($\bar{M}_n$) was calculated by multiplying $\overline{DP}$ (15.5) by repeating unit molar masses (HAPe1 = 335.42 g/mol x 15.5 = 5209 g/mol). The conversion of terminal alcohols into esters was determined by dividing the integration of the ester methylene peak (δ = 4.06; 2.5353H) by the sum of the integration of the methylene peaks adjacent to the ester and unreacted alcohol (δ = 3.65; 0.0352H + 2.5353H = 2.5705H; 2.5353H/2.5705H = 0.986 x 100% = 98.6%). The number of alcohols post-modified into fatty esters was determined by dividing the normalized integration of the sum of methylene next to ester and methylene next to alcohol (2.5705H/2H = 1.285) to the normalized integration of the methyl (1.285/0.07326 = 17.5). This value was multiplied by the percent of post-functionalization (P_f%; 17.5 x 98.6% = 17.29). The molecular weight contribution of the fatty esters was calculated by multiplying R by the molecular weight of C12 (183.32 g/mol x 17.29 = 3171 g/mol) or C16 (239.42 g/mol).

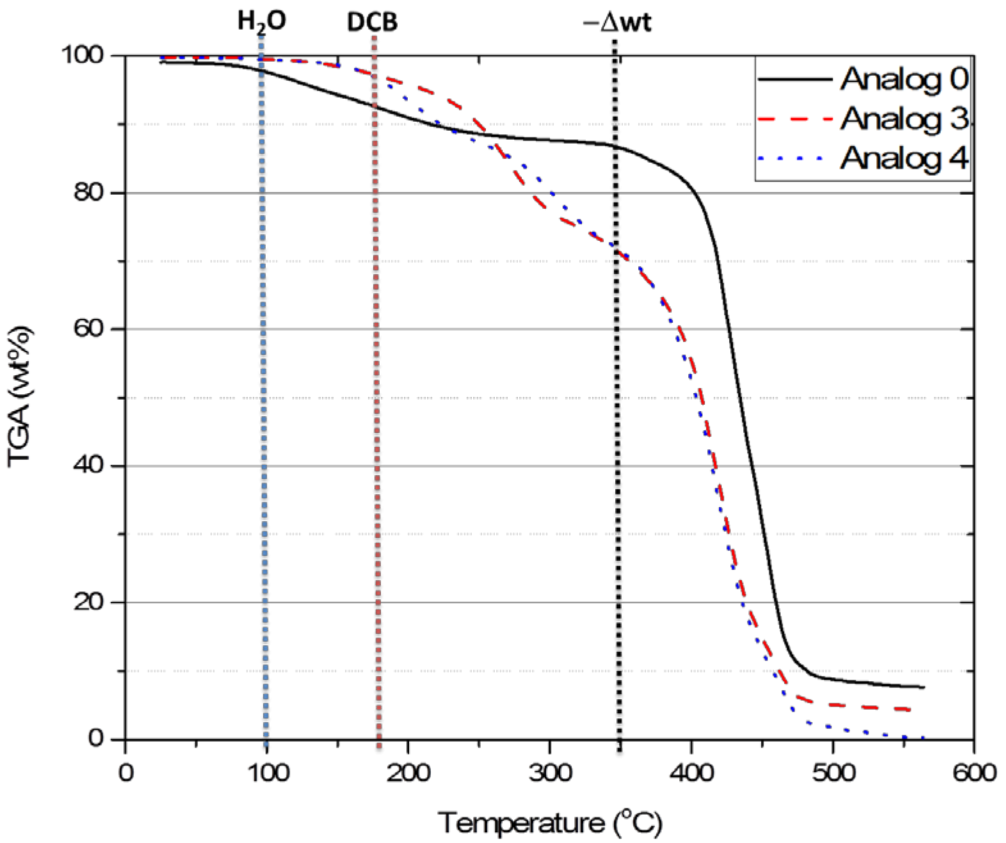

S6. Thermal Gravimetric Analysis of *HAPe2* prior to post-modification (Analog 0), Analog 3, and Analog 4. Initial mass losses are due to H_2O (bp = 100 °C) and dichlorobenzene (DCB; bp = 180 °C). Low molecular weight species are lost (Δwt = 10-25%) below and polymer mass losses occur at/above 350 °C.

S6. Internal tracking of samples.

Analog	Book and page	Date
1	61753-162	September 17, 2014
2	61753-161	September 17, 2014
3	61753-149	August 6, 2014
4	61753-148	August 6, 2014
5	61753-79	June 4, 2014
6	61753-69	May 14, 2014
7	61753-95	June 10, 2014
